# Primary Bone Lymphoma Masquerading as Multiple Myeloma: Challenges in the Diagnostic Workup of Severe Hypercalcemia

**DOI:** 10.7759/cureus.51856

**Published:** 2024-01-08

**Authors:** Elise Kahn, Laila Nomani, Alexandra M Harrington, Nisar Asmi

**Affiliations:** 1 Department of Internal Medicine, University of Colorado, Aurora, USA; 2 Department of Internal Medicine, Medical College of Wisconsin, Wauwatosa, USA; 3 Department of Pathology, Medical College of Wisconsin, Wauwatosa, USA; 4 Section of Hospital Medicine, Division of General Internal Medicine, Medical College of Wisconsin, Wauwatosa, USA

**Keywords:** lytic bone lesion, diagnostic anchoring, diagnosis of multiple myeloma, diffuse large b cell lymphoma (dlbcl), severe hypercalcemia

## Abstract

In this case, we explore the diagnostic workup of a patient presenting with symptomatic hypercalcemia. Initially suspected to have multiple myeloma, the diagnostic evaluation instead unveiled non-germinal center (non-GC) diffuse large B-cell lymphoma (DLBCL). DLBCL is the most common histologic subtype of non-Hodgkin lymphoma and is heterogeneous in terms of presentation, genetic drivers, and morphology. As primary bone DLBCL is exceedingly rare, the case presented proved to be a diagnostic challenge. The patient presented with one week of weakness, one to two days of nausea, and leg pain. On admission, hypercalcemia, renal failure, anemia, and lytic bone lesions were present and suggestive of multiple myeloma. However, serum protein electrophoresis and immunoglobulin levels did not fit the 2016 World Health Organization (WHO) diagnostic criteria for multiple myeloma. A negative bone marrow biopsy also argued against a diffuse plasma cell neoplasm. Finally, a biopsy from another bone lesion was diagnostic of DLBCL. This case discusses an unusual presentation of DLBCL.

## Introduction

This study discusses the workup of a patient presenting with symptomatic hypercalcemia. The clinical constellation of hypercalcemia, lytic bone lesions, renal failure, and anemia are common presentations of multiple myeloma. However, diagnostic workup revealed an atypical and uncommon presentation of diffuse large B-cell lymphoma (DLBCL), highlighting the importance of keeping a wide differential, even for seemingly typical presentations.

DLBCL is the most common histologic subtype of non-Hodgkin lymphoma (NHL), accounting for approximately 25% of NHL. DLBCL is heterogeneous in terms of morphology, genetic drivers, biological behavior, and presentation. The pathogenesis is complex and results from the malignant expansion of germinal or post-germinal B cells, diagnostically defined as germinal center B-cell-like (GCB) and non-GCB [1-3].

A typical presentation involves a rapidly enlarging nodal mass usually in the neck, abdomen, or mediastinum. However, in up to 40% of cases, the disease arises in extranodal extramedullary tissue, most commonly the stomach or gastrointestinal tract [4,5]. While the bone marrow is involved in up to 30% of cases with typical nodal involvement, primary lymphoma of the bone is a rare entity that accounts for less than 2% of all lymphomas in adults [6-9]. Patients with primary bone DLBCL are often younger (median age 56 years) and more commonly male [10,11], in contrast to the average age of DLBCL diagnosis, which is 66 years [12]. We report a case of an unusual presentation of DLBCL. 

## Case presentation

The patient, a 66-year-old male with a significant past medical history including coronary artery disease status post coronary artery bypass graft, hypertension, atrial fibrillation, and diabetes, presented to a community hospital emergency department with a one-week history of weakness, one to two days of nausea and emesis, and right thigh pain. He additionally reported polyuria, constipation, and thirst. The patient’s spouse also indicated that he had mild forgetfulness over the past few weeks, such as forgetting where things were.

The patient was hemodynamically stable, with a blood pressure of 131/62 mmHg (consistent with past blood pressures), a pulse of 90 beats per minute, an O_2_ saturation of 99% on room air, and a respiratory rate of 16 breaths per minute. The patient was afebrile. On physical exam, the patient was conversant but somewhat confused. The patient exhibited an irregularly irregular rhythm. Lungs were clear upon bilateral auscultation with no signs of respiratory distress.

The patient was transferred to our center, a tertiary care center, initially requiring ICU-level care for a grossly deranged metabolic profile. After successful stabilization, the patient was transferred to the medical floor for further workup.

Diagnostic workup 

On presentation, labs were remarkable for significant derangements including elevated creatinine (6.2 mg/dL; reference range 0.7-1.3 mg/dL), elevated calcium (15.1 mg/dL; reference range 8.6-10.2 mg/dL), and low hemoglobin (8.1 g/dL; reference range 13.7-17.5 g/dL). X-ray showed multiple lytic lesions in the right femur. Profound hypercalcemia and kidney injury prompted consults with hematology and nephrology specialists.

Initial workup for hypercalcemia included a low parathyroid hormone (PTH) level (8.5 pg/mL; reference range 15.0-72.0 pg/mL), decreased vitamin D 1,25-OH (5.8 pg/mL; reference range 19.9-79.3 pg/mL), and decreased vitamin D 25-OH (27 ng/mL; reference range 30-100 ng/mL). Furthermore, the PTH-related protein (PTH-rP) level was normal (2.6 pmol/L; reference range <4.2 pmol/L).

Additionally, kappa-free light chains were elevated (57.84 mg/L; reference range 3.3-19.4 mg/L), and lambda-free light chains were elevated (46.03 mg/L; reference range 5.7-26.30 mg/L). However, the kappa/lambda ratio was normal (1.26; reference range 0.26-1.65). A serum protein electrophoresis (SPEP) showed a faint monoclonal band of IgM kappa. The protein gap was less than 4, the 24-hour urine did not demonstrate paraprotein, and serum immunoglobulins were within normal limits.

**Figure 1:**
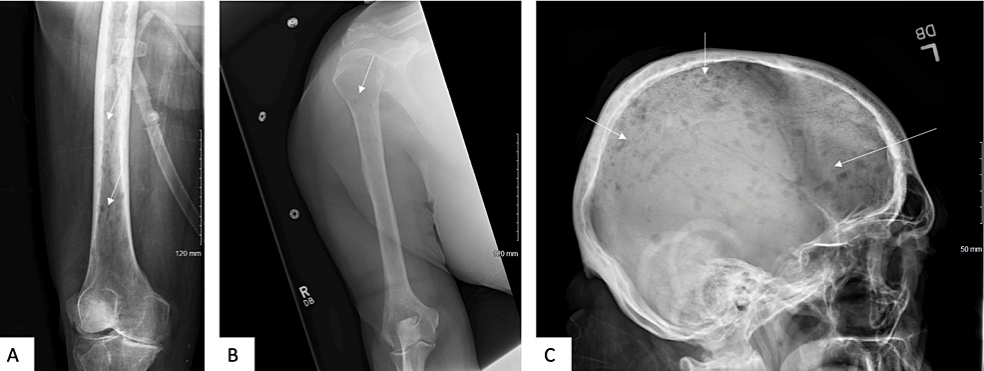
Skeletal survey demonstrated numerous lesions throughout the (A) femur, (B) humerus, and (C) skull.

A CT scan of the chest, abdomen, and pelvis revealed innumerable osseous lucencies throughout the skeleton, with no primary malignancy noted. A skeletal survey further demonstrated numerous lesions throughout the bilateral femurs, humerus, and skull (Figure 1).

Furthermore, lactate dehydrogenase (LDH) was only slightly increased (310 U/L; reference range 135-225 U/L); prostate-specific antigen (PSA), carbohydrate antigen 19-9 (CA 19-9), and carcinoembryonic antigen (CEA) were all within normal limits.

**Figure 2:**
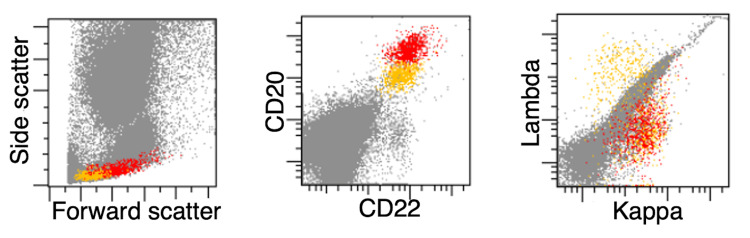
Bone marrow flow cytometry. The bone marrow flow cytometry plots demonstrate an abnormal, clonal B-cell population (red) with intermediate forward scatter, bright expression of CD20 and CD22, and kappa light chain restriction. This population was negative for CD5 and CD10 (not shown) and was admixed with normal polytypic B cells of small size (depicted in yellow). A clonal plasma cell population was not observed.

**Figure 3:**
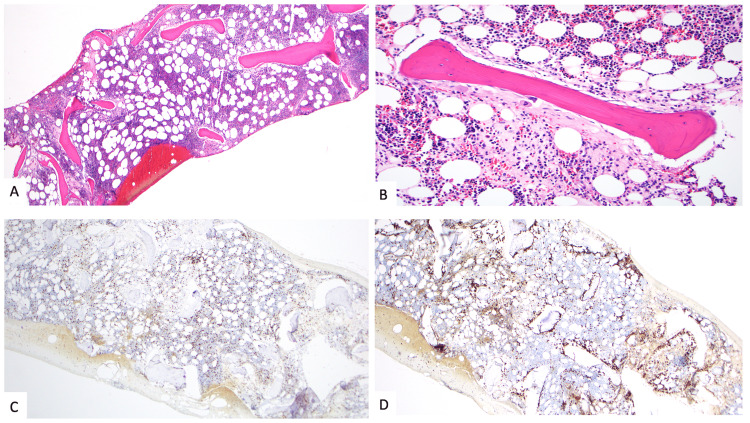
Bone marrow core biopsy histology. (A) The bone marrow core biopsy with aspiration demonstrates moderate hypercellularity with atypical paratrabecular pale areas (hematoxylin and eosin, 40x). (B) This area is composed of fibrosis and a spindled, small-sized lymphoid infiltrate (hematoxylin and eosin, 200x). Immunohistochemical stains for (C) CD3 (100x) and (D) CD20 (100x) demonstrate an atypical CD20(+) B-cell population arranged solely in a paratrabecular location throughout the entire core biopsy, supporting a diagnosis of lymphoma.

An iliac crest bone marrow biopsy was performed, which demonstrated paratrabecular infiltrates of lymphoma cells. Flow cytometric analysis identified a small clonal population of kappa-restricted CD5- and CD10-negative, intermediate-sized B cells (Figures 2-3).

**Figure 4:**
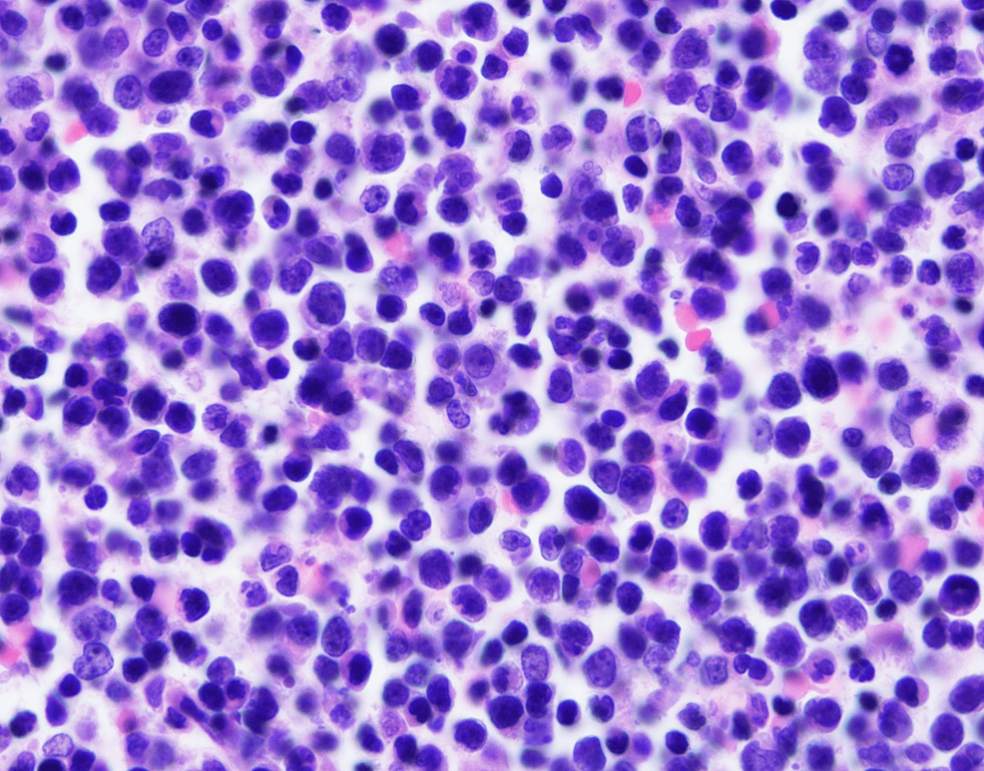
H&E (100x) biopsy of the right femur lytic lesion. The histologic section demonstrates an atypical lymphoid infiltrate comprising intermediate- to large-sized CD10-negative B lymphocytes expressing BCL6 and MUM-1, consistent with diffuse large B-cell lymphoma (DLBCL), activated B-cell type. H&E, hematoxylin and eosin

There was no evidence of plasma cell dyscrasia or metastatic carcinoma. A subsequent femur biopsy of a lytic lesion demonstrated DLBCL, activated B-cell type (Figure 4).

The low-grade B-cell lymphoma was believed to indicate to represent discordant bone marrow involvement.

Differential diagnosis 

Initial clinical presentation of hypercalcemia, anemia, renal failure, and lytic bone lesions all pointed toward a diagnosis of multiple myeloma. The initial diagnostic workup was consistent with multiple myeloma, including low PTH level, low vitamin D 1,25 OH, low vitamin D 25-OH, and low PTH-rP. Furthermore, SPEP demonstrated a faint monoclonal band of IgM kappa, and serum-free light chains were elevated, also suggesting a diagnosis of multiple myeloma.

Despite these clinical laboratory findings, the diagnosis of multiple myeloma was not confirmed as elevated free light chains is a nonspecific finding that can also be related to kidney injury or the result of another hematolymphoid proliferation. More importantly, the ratio between kappa and lambda was normal. Similarly, although electrophoresis revealed a faint band of IgM kappa, no evidence of plasma cell dyscrasia was observed on bone marrow biopsy. This makes multiple myeloma, light chain myeloma, and oligosecretory varieties of multiple myeloma less likely. Normal serum immunoglobulins and urine studies that did not reveal a clonal band provided further evidence against multiple myeloma.

Without a definitive diagnosis, further workup was performed, including a CT scan of the chest, abdomen, and pelvis to assess for PTH-rp associated malignancies that could cause a paraneoplastic syndrome with hypercalcemia, anemia, and renal failure. However, a CT scan did not demonstrate any primary malignancies, and PTH-rP was normal.

Bone marrow biopsy identified a CD5- and CD10-negative B-cell lymphoma. These diagnostic findings made the diagnosis of a solid malignancy with PTH-rP such as small cell carcinoma or prostatic carcinoma less likely and largely excluded the diagnosis of multiple myeloma; instead, diagnostic differential now included other hematolymphoid proliferations with aberrant IgM expression such as B-cell lymphomas, lymphoplasmablastic lymphoma, and hairy cell leukemia.

The patient then underwent a right femur biopsy of the lytic lesion. Histologic sections demonstrated an atypical lymphoid infiltrate comprising intermediate- to large-sized B lymphocytes expressing BCL6 and MUM-1, consistent with DLBCL, activated B-cell type. Once a lytic bone lesion biopsy demonstrated DLBCL, a diagnosis of stage IV-A non-GCB DLBCL was conclusively made.

Treatment

On presentation, the patient received intravenous (IV) fluids and calcitriol with meager improvements in calcium levels (decreased only to 14.7). Calcium levels were highest on admission at 15.1 and showed improvement with bisphosphonates, calcitonin, and IV fluids with concurrent diuretics. Due to significant renal failure and uremia, the patient was also treated with rasburicase.

R-CHOP (Rituximab, Cyclophosphamide, Doxorubicin, Vincristine, Prednisone) for six cycles is the standard initial treatment for DLBCL and was, therefore, administered.

Outcome

The patient was discharged to a subacute rehabilitation facility and subsequently transferred care to a local provider; therefore, all follow-up records are not available to us at this time. He continued with R-CHOP for the subsequent two cycles and then switched to an alternative chemotherapeutic regimen of R-GCVP (Rituximab, Gemcitabine, Cyclophosphamide, Vincristine, Prednisolone) due to the development of cardiac dysfunction. His clinical course was unfortunately further complicated by neutropenic sepsis. With continued functional decline, it was felt that further chemotherapy would not be appropriate, and he was ultimately transitioned to a comfort care pathway with hospice services at home. The patient ultimately passed away within six months.

## Discussion

The clinical diagnosis is determined through a combination of signs and symptoms within a relevant clinical context. Applying Bayes' theorem, clinicians assess the pretest likelihood based on the clinical setting and incorporate likelihood ratios for signs and symptoms observed during the clinical encounter to determine the posttest likelihood of a possible diagnosis. From a purely statistical perspective, a typical presentation of a common disease will have much higher odds of being the correct diagnosis when compared to an atypical presentation of a less common disease.

In the context of our case, a male patient in the seventh decade presenting with a symptom complex referable to the hematopoietic, renal, and musculoskeletal systems renders an intermediately high pretest probability of multiple myeloma. Positive likelihood ratios derived from a primary care cohort were 26 (95% confidence interval [CI] 18-35) for hypercalcemia, 5.3 (95% CI 5.0-5.7) for any cytopenia, 4.3 (95% CI 3.3-5.6) for bone pain, and 2.9 (95% CI 2.6-3.1) for elevated creatinine. When factored in, these values significantly increase the posttest probability for multiple myeloma in this scenario [13]. Such high estimates of posttest probability often cross diagnostic thresholds for most clinicians. In common clinical parlance, this estimated probability may be documented as *multiple myeloma unless otherwise demonstrated*.

Interestingly, the confirmatory testing in this case was statistically unexpected and turned out to be an atypical presentation of the less common disease. This a teaching opportunity, and one of the important reasons for this write-up is to advocate for keeping the list of possible differential diagnoses broad and guard against refuting possible differentials only based on statistical probabilities.

While multiple myeloma and lymphoma are both hematologic malignancies, they differ in their origins. Multiple myeloma originates from terminally differentiated plasma cells, while DLBCL is derived from B-cell precursor cells, not as far along the spectrum of differentiation.

According to the International Myeloma Work Group (IMWG) [14], the diagnosis of multiple myeloma requires meeting the following criteria: Clonal bone marrow plasma cells >10% or biopsy-proven bony or extramedullary plasmacytoma, along with one or more of the following: CRAB features (hypercalcemia, renal insufficiency, anemia, bone lesions), myeloma-defining events, including 60% or more clonal plasma cells on bone marrow examination, serum-free light chain ratio of 100 or greater, or more than one focal MRI lesion.

Because our patient presented with all of the CRAB features as well as some supporting investigations for multiple myeloma, the patient was initially presumed to have multiple myeloma.

On the other hand, DLBCL is ideally diagnosed from an excisional biopsy of a lymph node. In the event of cases such as the one discussed here, careful consideration must be taken to decide on the biopsy location if there is solely extranodal involvement [15]. It is not uncommon for DLBCL to demonstrate bone marrow involvement; however, primary bone DLBCL is exceedingly rare. The 2016 World Health Organization (WHO) classification of lymphoid neoplasms does not recognize primary bone marrow DLBCL as a separate entity [16].

Primary bone lymphoma is diagnosed once aggregates of malignant lymphoid cells are demonstrated to cause bone lesions without a concomitant lymph node involvement. Usually confined to the meta-diaphyseal region of long bones, most commonly the femur, it may involve multiple bones concurrently [17]. Radiologically, the lesions present as irregular, large lytic lesions that erode the cortex of the bone, sometimes extending into the adjacent soft tissue structures. Periosteal reactions remain rare [18]. Epidemiologically, primary lymphoma of bone is not a common disease. It constitutes only about 7% of all malignant bone tumors, 5% of all extranodal lymphomas, and less than 1% of all NHLs [19]. The pathogenesis of primary bone lymphomas is not fully understood; however, clinical and molecular data suggest that these tumors of the DLBCL type arise from the centrocytes [9].

There have been rare case studies documenting concurrent cases of multiple myeloma and DLBCL. These cases have been postulated to constitute a malignant transformation of lymphoma cells into plasma cells [17]. While this was not the case with our patient, further research into this field could shed light on the overlap of clinical presentation for the two malignancies.

## Conclusions

DLBCL is the most common histological subtype of NHL; however, primary bone DLBCL is rather rare. DLBCL is ideally diagnosed from an excisional biopsy of a lymph node; however, in the event of solely extranodal involvement, careful consideration must be taken to decide on a biopsy location.

This case highlights the importance of keeping a wide differential, even for seemingly typical presentations. The clinical workup of differential diagnosis should be based on their likelihood within the given context while being careful not to completely exclude any differentials based on low probability.
